# 预测贝伐珠单抗抗肿瘤治疗效果的生物学标记物

**DOI:** 10.3779/j.issn.1009-3419.2011.07.08

**Published:** 2011-07-20

**Authors:** 青青 潘, 孟昭 王

**Affiliations:** 100730 北京，中国医学科学院，中国协和医科大学，北京协和医院呼吸内科 Department of Respiratory Medicine, Peking Union Medical College Hospital, Chinese Academy of Medical Sciences and Peking Union Medical College, Beijing 100730, China

**Keywords:** 贝伐珠单抗, 生物学标记物, 肿瘤, 治疗, Bevacizumab, Biological markers, Neoplasms, Therapy

## Abstract

贝伐珠单抗已被用于多种恶性肿瘤的治疗，但仍没有一个公认的普遍适用的疗效预测指标。目前除了以影像学、副反应作为预测因子外，很多研究集中在对生物学标记物的筛选上。循环标记物中血管内皮生长因子（vascular endothelial growth factor, VEGF）水平对疗效的预测没有定论，而治疗前高水平可溶性血管内皮生长因子受体（soluble vascular endothelial growth factor receptor, sVEGFR）及低水平血管细胞间粘附因子-1、E-选择素、血管紧张素-2预示着治疗反应更好。肿瘤组织免疫组化方面，研究发现治疗前磷酸化VEGFR2与VEGFR2比值高或低表达碳酸酐酶9（carbonic anhydrase 9, CA9）、CD31-微血管密度（CD31-microvessel density, CD31-MVD）患者的疾病控制率高。另外基因方面的研究显示含有VEGF-634 CC和VEGF-1498 T者副反应较少，而携带VEGFR2 H472Q变异型基因者出现副反应的几率高。综上，要找到合适的生物学标记物来预测贝伐珠单抗抗肿瘤治疗的效果，需要进一步的基础研究去发现更特异的作用位点及更大规模的临床试验去验证。

## Abstract (en)

Bevacizumab, the monoclonal antibody of vascular endothelial growth factor (VEGF) has been applied to the therapy of several neoplasms, but an appropriate biomarker to predict the efficacy has not been found. Tose markers can originate from peripheral circulation, tumor tissue and genes. Some researches have found that low level of vascular cell adhesion molecule-1 (VCAM-1), E-selectin, angiopoietin 2 (Ang-2) in circulation or carbonic anhydrase 9 (CA9), CD31- microvessel density (CD31-MVD) in tumor tissue can predict beter activity of bevacizumab. Moreover, high level of soluble VEGFR2 (sVEGFR2) in circulation or the ratio of phosphorylated-VEGFR2 (p-VEGFR2) and VEGFR2 in tumor tissue increasing has the same predictive function. As to the gene, VEGF-634 CC, VEGF-1498 T and VEGFR2 H472Q are only related to the side effct. Thus more clinical tirals and basic researches should be performed to fnd out effective biomarkers in bevacizumab's therapy.

目前在抗肿瘤治疗中靶向治疗为研究的热门领域，其中抗血管内皮生长因子（vascular endothelial growth factor, VEGF）的单抗——贝伐珠单抗受到了极大的关注。几项大规模的临床试验^[1-4]^证明了贝伐珠单抗在抗肿瘤治疗中的有效性，如AVF2107、E3200、ECOG4599、AVAiL等，基于此，美国食品药品管理局已批准贝伐珠单抗与化疗药物联合使用于转移性结肠癌、非小细胞肺癌，在欧洲则被批准用于乳腺癌的治疗^[1]^。贝伐珠单抗用于这些患者的获益是明显的，其疗效评价和毒副反应如出血、血栓、高血压等是进一步研究的对象。由此引发一个问题，何种患者对贝伐治疗的治疗会呈现最好的疗效及最小的毒副反应？根据什么指标筛选这些患者？目前为止被研究的相关预测因子有很多，包括影像学检查、临床表现（如高血压）、皮肤病变^[5]^及生物学指标等。其中生物学指标通过血清、血浆或组织学标本检测可溶性及细胞表面标志物，并且分析RNA、DNA特征。由于其客观性和可操作性，具有重要的临床价值，也是本文主要讨论的对象。

## 1 VEGF通路介绍

病理状态下，VEGF在多种实体瘤及血液系统肿瘤中高表达，促进肿瘤血供和生长、眼内新生血管形成及参与炎症反应、促进水肿，其中对肿瘤发生发展的影响尤其引人关注^[6, 7]^。

在哺乳动物中VEGF生长因子家族包括VEGFA、VEGFB、VEGFC、VEGFD、胎盘生长因子（placenta growth factor, PIGF）。其中VEGFA基因包括8个外显子和7个内含子，经过不同剪切形成5种同工体VEGF145、VEGF165、VEGF121、VEGF189、VEGF206^[8]^。VEGF受体主要位于内皮细胞，部分位于骨髓细胞上，包括三种：VEGFR1，其配体为VEGFA、VEGFB、PIGF；VEGFR2，其配体为VEGFA、VEGFC、VEGFD、VEGFE；VEGFR3是VEGFC、VEGFD的受体，也是酪氨酸激酶受体中的一员，主要与淋巴管的形成有关。另外VEGFR家族还包括共受体神经纤毛蛋白、人硫酸肝素糖蛋白，能促进VEGFR2与VEGF165的结合，延长其半衰期^[9]^。

由于贝伐珠单抗只与VEGFA结合，而VEGFA的受体是VEGFR1、VEGFR2。因而这里主要介绍这两种受体及其信号传导途径^[10]^。除了血管内皮细胞，VEGFR1也在巨噬细胞、树突状细胞、成骨细胞表面表达。而VEGFR2还可表达在神经元等处。VEGFR2在胚胎早期表达增高，后期逐渐减低，并且在病理状态下如肿瘤中表达增高。

VEGFA与VEGFR1、VEGFR2结合后引起受体胞内段的交联、二聚化及酪氨酸激酶的激活，并导致不同细胞内作用位点的自身磷酸化，从而激活一系列下游的信号转导^[9]^。VEGFR2的激酶活性比VEGFR1高，与其它经典酪氨酸激酶受体如表皮生长因子受体（epidermal growth factor receptor, EGFR）、血小板衍生生长因子受体相当^[11-13]^。除了配体受体途径，有研究^[12, 14]^显示血流也可以直接激活VEGF系统而不依赖于配体。VEGFR2水平的下调对下游信号传导起负向调节作用。

根据VEGF通路，供检测的特异性生物学标志物按种类可分为：配体——VEGF（VEGFA）；受体——VEGFR1、VEGFR2、可溶性VEGF受体1（soluble vascular endothelial growth factor receptor 1, sVEGFR1）、sVEGFR2及下游代谢产物如磷酸化VEGFR1、2（phosphorylated-VEGFR1, p-VEGFR1; phosphorylated-VEGFR2, p-VEGFR2）、VEGFR的降解片段等；基因——VEGF、VEGFR的DNA、mRNA。标本来源包括血液（全血、血清或血浆）及肿瘤组织。同时其它与肿瘤血管生成相关的细胞因子也是被研究的对象。以下就将分别对其介绍。

## 2 循环中的生物标记物

贝伐珠单抗是针对VEGFA的重组人源化单克隆抗体，因而很多研究把血清或血浆中的VEGF作为研究对象。Duda^[15]^、Baar^[16]^观察到贝伐珠单抗治疗后血浆VEGF呈增高趋势，而Smerdel^[17]^则认为治疗后血清VEGF较前降低。有5项临床试验（Goede、Burstein、Cohen、Horn、Han）^[18-22]^得出的结论是治疗前的VEGF水平与治疗反应无关，这里包括了对直肠癌、乳腺癌、头颈部鳞癌、小细胞肺癌和卵巢癌等多种恶性肿瘤的临床研究。只有Dowlati等^[3]^在对非小细胞肺癌的ECOG4599和Smerdel等^[17]^对多重耐药卵巢癌的2项研究中发现治疗前VEGF水平与治疗反应有关。前者认为VEGF水平较高者对包含贝伐珠单抗的治疗反应更好，而后者的结果却刚好与其相反。需要注意的是以上各项试验均只研究了治疗前血清或血浆VEGF水平与治疗反应之间的关系，没有进一步研究治疗后VEGF变化趋势与疗效之间是否有联系。也有研究者检测尿液中VEGF，Stopeck等^[23]^认为治疗后尿液中VEGF轻度下降。虽然治疗前尿液中VEGF与治疗反应没有联系，但其水平较低者的疾病无进展时间（progression-free time, PFS）和总生存时间（overall survival, OS）却更长。

除了直接检测VEGF水平，也有关于其可溶性受体的相关研究。Smerdel等^[17]^研究发现，治疗后sVEGFR1无明显变化，而sVEGFR2在第二个疗程后有所升高。Duda等^[15]^进一步以127 pg/mL为界，将治疗前血浆sVEGFR1水平分为高低两组，结果发现高水平sVEGFR1者不仅治疗反应好，而且发生Ⅲ°以上不良反应的几率也小。

对于其它循环生物学标记物，由于贝伐珠单抗的抗血管生成作用，常被用于检测的也是一些与血管生理病理有关的细胞因子，如血管细胞间粘附因子-1（vascular cell adhesion molecule-1, VCAM-1）、细胞间粘附因子-1（intercellular adhesion molecule 1, ICAM-1）、血管紧张素-2（angiopoietin 2, Ang-2）、E-选择素（E-selectin）等。Baar^[16]^和Stopeck等^[23]^均发现治疗后VCAM-1升高，但其变化与治疗中是否包含贝伐珠单抗无关。另外Goede^[18]^、Baar等^[16]^认为治疗前这些细胞因子，如VCAM- 1、E-selectin、Ang-2，其水平较低者对治疗的反应更好。而对ICAM-1则没有得出类似结论，只是发现低水平ICAM-1者接受贝伐珠单抗治疗后疾病的进展风险也低^[3]^。

除了细胞因子，Ronzoni等^[24]^还研究了结直肠癌患者全血里的循环内皮细胞（circulating endothelial cells, CECs），其中包括总循环内皮细胞（total circulating endothelial cells, tCECs）、静息态（resting CECs, rCECs）或激活态（activated CECs, aCECs）循环内皮细胞以及循环内皮祖细胞（endothelial progenitor cells, CEPs）。结果发现与治疗反应有关的是tCECs和rCECs，达到完全或部分缓解者的tCECs降低，而疾病无进展或部分进展的患者rCECs升高。

## 3 组织中的生物标记物

与贝伐珠单抗治疗有关的组织学标记物，最常被研究的是VEGF及其受体VEGFR。Sathornsumetee等^[25]^对恶性胶质瘤患者进行了研究，结果显示治疗前肿瘤组织高表达VEGF者，从影像学上观察到的治疗反应更好。然而在对结直肠癌和复发耐药卵巢癌患者的相关研究中，却没有发现这一关系。Hong、Han、Wedam等^[22, 26, 27]^进一步研究了治疗后VEGF的变化趋势与疗效的关系，结果发现对于乳腺癌患者，治疗有反应者VEGF下降的更明显。这些结论的不一致，其中原因一是VEGF在肿瘤组织中的分布不均一；二可能与取材有关，如果是通过活检方法获得的标本，则其获得的肿瘤组织量有限，代表性不够；三是VEGF的基础表达值与治疗后的变化情况对疗效的反应程度不同。

VEGF通路上除了VEGF，另一个关键点是VEGF受体。Wedam等^[27]^认为治疗前VEGFR水平与治疗反应无关，却发现治疗后VEGFR的活化状态——p-VEGFR在部分缓解或疾病无进展的患者中呈下降趋势，而在疾病进展者中升高。Cohen^[20]^也把p-VEGFR作为研究靶点，不过其观察的是治疗前p-VEGFR2与VEGFR2的比值，并得出其比值越高，治疗后肿瘤缩小率更高的结论。

其它组织学标记物还有低氧诱导因子2α（hypoxiainducible factor-2α, HIF-2α）、碳酸酐酶9（carbonic anhydrase 9, CA9）及CD31-微血管密度（CD31-microvessel density, CD31-MVD）。相关的研究里，Wedam^[27]^、Sathornsumetee^[25]^没有发现以上这些标记物与治疗反应有关。Hong^[26]^、Han^[22]^则分别在结直肠癌和卵巢癌患者中发现，低表达CA9的患者疾病控制率高，而高水平CD31-MVD预测着较差的治疗反应。CD31-MVD直接反映肿瘤血管增生情况，而CA9作为HIF-2α的下游因子，在肿瘤血管增生的诱因——缺氧时表达增高。因而两者的研究结论共同说明肿瘤血管增生明显，则治疗反应下降。

## 4 基因相关的生物标记物

相关标记物的基因，标本来源可以是外周血也可以是肿瘤组织。Smerdel^[17]^检测了治疗前外周血中VEGF的DNA，结果发现VEGF-2578、-1154、-460、+405、+936这五个位点无论是与治疗反应还是与生存指标都没有关系。而ECOG2100和BOND-2^[28, 29]^的标本都来自肿瘤组织，前者分析了乳腺癌组织中*VEGF*基因的多样性，后者检测了结直肠癌组织中*VEGFR2*基因的含量。结果两者都没有找到基因与治疗反应间的关系，却发现含有VEGF-2578 AA、VEGF-1154 AA或VEGFR2高表达者OS更长。进一步研究还发现含有VEGF-634 CC和VEGF-1498 TT者，出现毒副反应的几率也较低。与此类似，Jain^[5]^也找到基因与治疗副作用之间的联系，其对多种实体肿瘤的VEGFR2基因做了研究，结论是携带VEGFR2 H472Q变异型基因者发生高血压、手足皮肤病变的几率高。虽然副作用不是预期疗效的一部分，但也可以间接反映出治疗是否起效，所以可以通过试验，进一步去寻找基因与副作用或疗效之间的关系。

## 5 总结

通过以上总结（临床试验具体内容见表 1）可以发现，对于预测贝伐珠单抗的抗肿瘤治疗效果，目前还没有一个确实有效的预测标记物，无论是配体VEGF，还是与低氧相关的HIF-2α、CA9或与下游反应有关的VEGFR、CECs。但是如果标记物与VEGF通路的关系更特异，如活化的VEGFR——p-VEGFR，或联合多个指标共同预测，比如p-VEGFR2/VEGFR2、CD31-MVD，预测的有效性有所提高。另外除了检测各个标记物的基础水平，还可以根据其治疗后的变化趋势来预测治疗是否有效。

**1:** 相关临床试验：与预测贝伐珠单抗抗肿瘤治疗效果有关的生物学标记物 Clinical trials about predictive biomarkers for bevacizumab in anti-tumor therapy

研究者	研究 例数	肿瘤名称	治疗方案	所测生物学标记物	试验结果
BOND-2^[29]^	83	伊立替康难治性转移性结直肠癌	西妥昔单抗+贝伐珠单抗+伊立替康/西妥昔单抗+贝伐珠单抗	微切片肿瘤组织测表皮生长因子受体（epidermal growth factor receptor, EGFR）、血管内皮生长因子（vascular endothelial growth fac tor, VEGF）、血管内皮生长因子受体2（vascular endothelial growth factor receptor 2, VEGFR2）的DNA、RNA、cDNA或RT-PCR测量mRNA的表达水平	（1）肿瘤组织EGFR高表达； （2）VEGFR2与总生存时间（overall survival, OS）延长有关，其它基因与生存指标无关。
Jain^[5]^	178	各种实体肿瘤（非小细胞肺癌、前列腺癌、结直肠癌、卡波西肉瘤）	贝伐珠单抗（15 mg/kg或5 mg/m^2^）、索拉非尼、沙利度胺、西妥昔单抗、蛋白酶抑制剂	PCR法测血浆或全血VEGFR2基因是否存在H472Q或V297I变异型	携带VEGFR2 H472Q变异型基因者发生高血压（26例）、手足皮肤病变（22例）的几率高（高血压在变异型和野生型中的发生率分别为39%和21%，*P*=0.015；手足皮肤病变的发生率分别为33%和16%，*P*=0.013）。
Duda^[15]^	32	局部进展的直肠癌	贝伐珠单抗单药（5 mg/kg）序贯贝伐珠单抗+ 5-氟尿嘧啶（5-Fu）及放疗	测治疗前及治疗后第1、3、1 2、32、9 6天的血浆和尿中的V E G F、胎盘生长因子（placenta grow th fac tor, PIGF）和可溶性VEGF受体1（soluble vascular endothelial growth factor receptor 1, sVEGFR1）、sVEGFR-2的含量	（1）VEGF、PlGF在治疗后各检测点均升高；sVEGFR2仅在第3天升高；sVEGFR1仅在第3天下降；尿中各指标未见改变； （2）治疗前高水平血浆VEGFR1（切点127 pg/mL）与治疗反应好有关（Mandard score评分高，*P* < 0.01）； （3）治疗前高水平血浆VEGFR-1，发生Ⅲ°不良反应率为0。
Goede^[18]^	123	结直肠癌	贝伐珠单抗+化疗药	测治疗前血清血管紧张素- 2（angiopoietin 2, Ang-2）的含量（切点3.5 ng/mL）、VEGF（切点0.19 ng/mL）	（1）结直肠Ⅳ期患者的Ang-2水平比Ⅰ期-Ⅲ期和正常人高； （2）治疗前A n g - 2水平较低的患者治疗反应较好 （*P* < 0.01)、疾病无进展时间（progression-free time, PFS）长（*P* < 0.01)； （3）治疗前VEGF水平与治疗反应、PFS无关。
Ronzoni^[24]^	40	晚期结直肠癌	贝伐珠单抗（第1、2周：5 mg/kg，第3周：7.5 mg/ kg）+化疗药	流式细胞仪法测第一、三、六疗程治疗前总循环内皮细胞（total circulating endothelial cells, tCECs）、静息态（resting CECs, rCECs）和激活态（activated CECs, aCECs）循环内皮细胞以及循环内皮祖细胞（endothelial progenitor cells, CEPs）的数量	（1）结直肠癌患者中tCECs、rCECs升高明显； （2）影像学上完全或部分缓解的患者治疗后tCEC s降低（*P*=0.02）； （3）影像学上疾病进展或稳定的患者rCECs升高（*P*=0.07）。
Hong^[26]^	31	治疗过的转移性结直肠癌	贝伐珠单抗（第1、2周：5 mg/kg，第3周：7.5 mg/kg）+化疗药	免疫组化法测肿瘤组织VEGF（切点80积分）、碳酸酐酶9（carbonic anhydrase 9, CA9）表达水平	（1）低表达CA9的患者疾病控制率高、疾病无进展时间和总生存时间长； （2）VEGF与治疗反应或生存指标无关（高表达组与低表达组的总反应率分别为62.5%和60.0 %，*P*=0.919）。
Wedam^[27]^	21	炎性和局部进展的乳腺癌	贝伐珠单抗（15 mg/kg）+多西紫杉醇+多柔比星	免疫组化法测治疗前后肿瘤组织V EG F、微血管密度（microvessel density, MVD）、VEGFR2、磷酸化VEGFR2（phosphorylated-VEGFR2, p-VEGFR2）（Y951、Y996）表达水平	（1）贝伐治疗后p - VEGFR2（Y951、Y996）明显下降（*P*=0.004, *P*=0.025）； （2）部分缓解和疾病稳定的患者治疗后p-VEGFR2（Y951、Y996）下降，疾病进展的患者p-VEGFR2（Y951、Y996）升高； （3）治疗有反应者VEGF下降更明显（治疗有反应者和无反应者的下降率分别为57.6%和0）； （4）MVD、VEGFR2、p-VEGFR2与治疗反应无关。
Baar^[16]^	49	局部进展的乳腺癌	贝伐珠单抗（10 mg/kg）+多西紫杉醇（DB组）和多西紫杉醇（D组）	测治疗前、第8周、第17周、第3、6、12个月血浆VEGF及血清E-选择素、血管细胞间粘附因子-1（vascular cell adhesion molecule-1, VCAM-1）、细胞间粘附因子-1（intercellular adhesion molecule 1, ICAM-1）含量	（1）第17周DB组VEGF、VCAM-1水平比D组上升的明显（VEGF: *P* < 0.0001; VCAM-1: *P*=0.069）； （2）ICAM升高（*P* =0.018）、E-选择素降低（*P* =0.006），但DB组和D组无差别； （3）单因素模型分析示治疗前VCAM-1、E-选择素水平低的治疗反应好（*P*=0.033, *P*=0.035）。
ECOG2100^[28]^	363	晚期乳腺癌	贝伐珠单抗+/-紫杉醇	RT - PCR法对肿瘤组织标本中的VEGF、VEGFR2进行寡核苷酸基因多态性的分析	（1）VEGF-2578 AA、VEGF-1154 AA与总生存时间增加 （*P*=0.023, *P*=0.001）； （2）VEGF-634 CC和VEGF-1498TT与较低的毒副反应有关（*P*=0.005, *P*=0.022）。
Burstein^[19]^	56	Ⅳ期乳腺癌	贝伐珠单抗（10 mg/kg）+长春瑞滨	治疗前测血浆VEGF（切点32.6 pg/ mL）含量	（1）高低水平组VEGF与治疗反应无关； （2）VEGF水平低的至疾病进展时间（time to progression, TTP）延长。
Cohen^[20]^	48	头颈部转移性鳞状细胞恶性肿瘤	贝伐珠单抗（15 mg/kg）+厄洛替尼	治疗前免疫组化法测肿瘤标本的VEGFR 2、pVEG FR 2、EGFR、pEGFR；同时测血清中VEGF（切点52 p g /mL）和转化生长因子-*α*（transforming growth factor – *α*, TGF-*α*），切点28.9 pg/mL）表达水平	（1）完全缓解者（2例）与部分缓解、疾病稳定、部分进展者（9例）相比：p-VEGFR2/VEGFR2、p-EGFR/ EGFR比值更高（*P*=0.036; *P*=0.036）且肿瘤缩小率更高（*P*=0.007; *P*=0.008）； （2）血清VEGF、TGF-*α*的水平与治疗反应、PFS、OS无关。
Sathornsumetee^[25]^	60	恶性胶质瘤	贝伐珠单抗+厄罗替尼+/-抗癫痫药	免疫组化法测肿瘤组织中的VEGF-A、VEGFR2、CA9、CD31、低氧诱导因子2*α*（hypoxia-inducible factor-2*α*, HIF-2*α*）表达水平	（1）高表达VEGF的患者（共20例）影像学治疗反应更好（完全缓解和部分缓解者为9例，*P*=0.024）； （2）CD31、CA9和HIF-2*α*与治疗反应无关； （3）VEGFR2表达较低的患者的1年生存率较高（*P*=0.008）。
Stopeck^[23]^	52	复发进展的非霍奇金淋巴瘤	贝伐珠单抗（10 mg/kg）+化疗药	测治疗前、第8周、全部治疗后血浆VCAM-1（切点789 ng/mL）、尿中的VEGF（切点68 pg/mL）、全血中的CEC（切点33 CEC /uL）含量。免疫组化测治疗前骨髓活检标本中的VEGF、VEGFR1、VEGFR2表达水平	（1）治疗后尿液VEGF下降（由67 pg/mL降至51 pg/ mL）、血浆VCAM上升（由750 pg/mL升至1, 142 pg/ mL）、CEC下降（由81 CEC/uL降至22 CEC/uL）； （2）治疗前尿液VEGF、血浆VCAM低的PFS、OS更长（PFS: *P*=0.05, *P*=0.03; OS: *P*=0.07, *P*=0.06）； （3）治疗前CEC水平低的12例患者中对治疗有反应者治疗后CEC水平升高； （4）免疫组化中VEGF、VEGFR1主要表达于肿瘤细胞表面；VEGFR2主要表达与内皮细胞表面。
ECOG4599^[3]^	878	非鳞癌的非小细胞肺癌	B P C组：紫杉醇+卡铂+贝乏单抗（15 mg/kg）；PC组：紫杉醇+卡铂	治疗前血浆VEGF（切点值35.7 pg/ mL）； 治疗前及第7周血浆成纤维细胞生长因子（basic fibroblast growth factor, bFGF）、ICAM（切点260 ng/mL）、E-selectin的含量	（1）高水平VEGF：BPC组的有效率（33%）高于PC组（7.7%），*P*=0.01； （2）低水平VEGF：PC组和BPC组有效率分别29%和28.6%，*P*值无意义； （3）治疗前血浆ICAM低者中，PC组/BPC组有关PFS的风险比（hazard ratio, HR）为2.14。
Horn^[21]^	63	治疗过晚期小细胞肺癌	贝伐珠单抗（15 mg/kg）+顺铂+依托铂苷	治疗前、第7周测血浆VEGF（切点52 pg/mL）、ICAM（291 ng/mL）、VCAM（626 ng/mL）、E-选择素（32 ng/mL）、bFGF（5.01 pg/mL）的含量	所有标记物与治疗反应无关
Han^[22]^	62	复发耐药的卵巢癌及腹膜肿瘤	贝伐单药（15 mg/ kg）	治疗前及第4程前免疫组化法测肿瘤组织CD31-MVD（切点14）、VEGF（切点10 0）、p53表达水平和血浆及血清VEGF的含量（切点值分别为79.95 pg/mL和445.92 pg/mL）	（1）治疗前血浆、血清VEGF水平高低与治疗反应、PFS无关； （2）治疗前高水平血清VEGF者的中位生存期缩短22个月、死亡风险增高(HR=2.666)； （3）治疗前CD31-MVD水平高者对治疗反应差，高表达CD31-MVD者治疗反应率为14%（3/22），低表达CD31-MVD治疗反应率39%（7/18）； （4）治疗前VEGF、p53表达水平与治疗反应无关； （5）p53表达阳性的女性患者总生存时间长。
Smerdel^[17]^	38	多重耐药卵巢癌	贝乏单抗单药（10 mg/kg）	每程治疗前测血清VEGF（切点值540 pg/mL）、VEGFR1-2（浓度分别为64 pg/mL-222 pg/mL和4, 895 pg/mL-17, 760 pg/mL）及CA125的含量，同时PCR法测全血VEGF-DNA多态性	（1）治疗后血清VEGF下降；sVEGFR1无明显变化；sVEGFR2在第二个疗程后升高； （2）治疗前低VEGF水平组（15例）治疗反应率更高为60%，*P*=0.01； （3）VEGF-2578、-1154、-460、+405、+936及血清VEGFR1-2与治疗效果或PFS、OS均无关。

除了目前临床试验中常用到的生物学标记物，有些基础试验还通过机制研究，发现了一些新的标记物，如Zhang^[10]^在一项离体试验中发现，正常组织低表达VEGFR1、高表达VEGFR2，鳞状细胞癌的血管内皮细胞上却高表达VEGFR1、低表达VEGFR2，则推测贝伐珠单抗的作用应该是使VEGFR的表达“正常化”，即拮抗肿瘤组织产生过多的VEGF配体，继而使肿瘤组织中的VEGFR1、VEGFR2表达“翻转”。其还发现VEGF激活下游通路的过程中会降解VEGFR2，产生一种160 kDa新的多肽片段。如果能在临床上检测这些指标，研究其与贝伐珠单抗治疗效果的关系，可能会更有助于找到更方便、确切及高效的生物学标记物。
